# A New Highly Bioactive Composite for Scaffold Applications: A Feasibility Study

**DOI:** 10.3390/ma4020339

**Published:** 2011-01-28

**Authors:** Devis Bellucci, Valeria Cannillo, Antonella Sola

**Affiliations:** Dipartimento di Ingegneria dei Materiali e dell’Ambiente, Università degli Studi di Modena e Reggio Emilia, Via Vignolese 905, 41125 Modena, Italy; E-Mails: devis.bellucci@unimore.it (D.B.); antonella.sola@unimore.it (A.S.)

**Keywords:** composites, hydroxyapatite, bioactive glasses, scaffolds

## Abstract

Hydroxyapatite (HA) has been widely investigated as scaffolding material for bone tissue engineering, mainly for its excellent biocompatibility. Presently, there is an increasing interest in the composites of hydroxyapatite with bioactive glasses, with the aim to obtain systems with improved bioactivity or mechanical properties. Moreover, modifying the ratio between bioactive glass and hydroxyapatite results in the possibility of controlling the reaction rate of the composite scaffold in the human body. However, high temperature treatments are usually required in order to sinter HA-based composites, causing the bioactive glass to crystallize into a glass-ceramic, with possible negative effects on its bioactivity. In the present research work, a glass composition belonging to the Na_2_O-CaO-P_2_O_5_-SiO_2_ system, with a reduced tendency to crystallize, is applied to realize HA-based composites. The novel samples can be sintered at a relative low temperature (750 °C) compared to the widely studied HA/45S5 Bioglass^®^ composites. This fact greatly helps to preserve the amorphous nature of the glass, with excellent effects in terms of bioactivity, according to *in vitro* tests. As a first application, the obtained composites are also tested to realize highly porous scaffolds by means of the standard burning out method.

## 1. Introduction 

Bone tissue engineering [1,2,3] is an emerging biomedical technology aiming to develop alternative protocols for bone tissue repair and regeneration. In a typical application, tissue is harvested from the patient and then it is dissociated into individual cells using enzymes. The cells are then seeded on a scaffold [4,5,6], *i.e*., a proper porous structure which acts as a guide for tissue re-growth in three dimensions, obtaining a living bio-composite system. From this point of view, scaffolds are artificial structures designed as temporary templates for cell adhesion and proliferation. The damaged tissue is removed from the patient body and the tissue/scaffold composite is reimplanted in the defect site of the patient, where the scaffold will be resorbed while the cells are producing their own natural extracellular matrix. To be useful, scaffolds should be highly bioactive and mimic the human bone morphology, which is highly porous with an interconnected pore network [7,8,9]. The pore structure is necessary for the infiltration of cells into the scaffold, for the supply of nutrients and fluids, and the washout of cell wastes. The scaffold material should also be able to promote cell adhesion and, ideally, at least to stimulate osteogenesis at the genetic level [10,11]. At the same time, adequate mechanical properties are required for scaffolds, in order to match those of the tissue to be restored. Moreover, it is highly desirable to optimally control the reaction rate of the scaffold in the human body, which is expected to range from a slightly bioactive behavior to a quick and complete bioresorption with appropriate degradation properties depending on the specific application [12,13].

In the last years, scaffolds based on synthetic calcium phosphate bioceramics, such as β-tricalcium phosphate (β-TCP) and, in particular, hydroxyapatite (HA), have been realized by means of different techniques [14,15]. The main advantage of HA (Ca_10_(PO_4_)_6_(OH)_2_) is its chemical similarity to the mineral component of natural bone, which promotes the ability to form a bond with the surrounding bone tissue when implanted. For these reasons, HA gained acceptance as a bone substitute and it is nowadays employed in oral, maxillofacial and orthopaedic applications [16,17,18]. On the other hand, even if the HA biocompatibility is excellent, its reactivity with the existing bone is low, therefore the rate at which the bone integrates with HA is relatively low [19]. These facts can be detrimental for scaffold applications.

There have been many attempts to combine HA with other bioceramics, in order to produce composite materials with improved biological properties [20,21]. Recently, the interest of many material scientists has been focused on the possibility to sinter HA with bioactive-glass addictions. The particular composition of bioactive glasses (or bioglasses), originally discovered by Hench and coworkers at the end of the 1960s, makes them highly reactive when in contact with body fluids. As a consequence, bioglasses are able to build tenacious bonds with the surrounding bone, through the formation of a surface layer of hydroxy-carbonate apatite (HAC) which drives and promotes osteogenesis, allowing the rapid formation of new bone [22,23]. Compared with bioactive glasses, HA and β-TCP are characterized by more bland surface reactions and minimal ionic release [19]. The addition of such glasses to HA is expected to improve not only the bioactivity of the system, but also its sinterability, since bioactive glasses typically melt at a lower temperature compared to HA; the glassy phase, when incorporated into HA in order to obtain composite materials, enhances the densification and the resulting mechanical strength.

Several bioglasses of different compositions have already been tested in HA-based composites. Many of them belong to the Na_2_O-CaO-P_2_O_5_ or CaO-P_2_O_5_-SiO_2_ systems [24,25,26,27]. In particular the 45S5 Bioglass^®^ [22], whose reference proportions (46.1 mol % SiO_2_, 26.9 mol % CaO, 24.4 mol % Na_2_O and 2.6 mol % P_2_O_5_) make it able to bond to both hard and soft tissues, has been used [28].

On the other hand, among the disadvantages of using bioglasses in HA-based composites, it should be noted that, in spite of the sintering aid exerted by the glass, high-temperature treatments are often required in order to sinter these systems. The usual sintering processes, performed in a range of temperatures between 1200 °C and 1300 °C, cause the HA to decompose, with subsequent formation of tricalcium phosphate or CaO [29]. Additionally, at such temperatures, reactions occur between the HA and the glass, which provoke a reduction in the HA amount, an alteration of the lattice parameters of the residual HA, and the development of new phases, such as α- and β-tricalcium phosphate (TCP) [30].

The presence of α- or β-TCP should be avoided, since it alters the biodegradability of the final system. On the other hand, CaO and the subsequent formation of Ca(OH)_2_ may cause decohesion of the composite, because of the stresses due to volume changes [31]. It should also be noted that high temperature treatments may cause the bioactive glass to crystallize into a glass-ceramic prior to complete densification, with possible negative effects on its bioactivity [32]. The bioactivity of these materials, in fact, is based both on their composition and on their inherently amorphous nature, which facilitates the solubility both *in vitro* and *in vivo*. These considerations, among others, initially led materials scientists to look with pessimism on the feasibility of highly porous bioglass-based scaffolds. For example, Li [32] and coworkers reported a HAC layer formation *in vitro* only with a high proportion (over 90%) of glassy phase in the glass-ceramic sample. However, the findings of various studies on 45S5 Bioglass^®^-derived glass ceramics show that crystallization decreases the kinetics of the HAC layer formation without inhibiting the development of such a layer [4,33]. For these reasons, the development of glass compositions with a reduced tendency to crystallize is desired for the production of scaffolds and, in general, HA-based composites, since the rate of HAC formation and, presumably, the system bioactivity, decrease when the percentage of crystallization increases [33,34]. 

In this work, a specific glass composition (BG_Ca), whose proportions are 47.3 mol % SiO_2_, 45.6 mol % CaO, 4.6 mol % Na_2_O and 2.6 mol % P_2_O_5_, is applied to realize HA-based composites (HA/BG_Ca) [35]. This glass shows a low tendency to crystallize compared to the widely used 45S5 Bioglass^®^, which belongs to the same Na_2_O-CaO-P_2_O_5_-SiO_2_ system. Most of all, BG_Ca pressed powders are characterized by the ability to consolidate at rather low temperatures (750 °C), before the crystallization starts, with positive effect on the resulting bioactivity. It should be noted that such a heat treatment results in a cheaper technological protocol; on the other hand, it is possible to prevent the hydroxyapatite decomposition, which typically occurs at higher temperatures. 

In the first part of this feasibility study, the bioactivity of the novel HA/BG_Ca samples was tested in a simulated body fluid solution (SBF) [36]. Afterwards, HA/BG_Ca powders were used to produce composite scaffolds by means of the polymer burning out method [37,38]. This technique, which is probably the simplest way to generate porous scaffolds from ceramics, employs organic fillers as pore generating agents in a ceramic matrix. The organic phase is added to the ceramic powders and then it is thermally removed during sintering. This method has the advantage of combining versatility and low cost. The obtained scaffolds were analyzed from a microstructural point of view, paying particular attention to their porosity (content and morphology). The realized scaffolds possessed a good manageability and an adequate porous structure, together with an appreciable mechanical behavior, according to compression tests.

## 2. Materials and Methods

### 2.1. HA/BG_Ca Composites Preparation

The BG_Ca was prepared by melting the raw powder materials (commercial SiO_2_, CaCO_3_, Ca_3_(PO_4_)_2_, Na_2_CO_3_ by Carlo Erba Reagenti, Italy) in a platinum crucible. The following thermal cycle was set: from room temperature to 1100 °C at 10 °C /min; at 1100 °C for 1 h; from 1100 °C to 1450 °C at 10 °C /min; at 1450 °C for 30 min. Then the melt was rapidly quenched in water. The obtained frit was dried overnight in a furnace at 110 °C, ball-milled and finally sieved to a grain size below 38 μm. 

BG_Ca powders were mixed by 50 wt % with HA powders to obtain the HA/BG_Ca composite. Commercial HA (CAPTAL^®^ Hydroxylapatite, Plasma Biotal Ltd, U.K.), with an average particle size below 25 μm, was used. HA/BG_Ca powders were mixed for 6 h in a plastic bottle using a rolls shaker in order to obtain an effective mixing. Then the powders were used to produce green bodies by uniaxial pressing at 140 MPa for 10 s using propanol as a liquid binder. The pressed samples, shaped in form of disks (4 cm of nominal diameter, 0.7 cm of thickness), were heat-treated for 3 h in a furnace. Various sintering temperatures were considered and the densification of the composites was monitored by measuring the Archimedean density and the volume shrinkage. The thermal treatment was set at a final temperature of 750 °C with a 5 °C/min heating rate.

### 2.2. Scaffolds Fabrication

The scaffolds were prepared by mixing the HA/BG_Ca powders with a thermally removable organic phase acting as a pore generating agent. With this aim, polyethylene (PE) powders (Goonvean Fibres, U.K.) mixtures with the following composition were employed: 80 wt % PE powders with particle size within 90–150 μm and 20 wt % PE powders with particle size within 300–500 μm. 50% vol. of HA/BG_Ca powders were added to 50% vol. of PE powders, and the obtained mixture was further mixed for 30 min. in a plastic bottle using a rolls shaker. Then the powders were used to produce green bodies by uniaxial pressing at 140 MPa for 10 s using propanol as a liquid binder. The pressed samples, shaped in form of disks (4 cm of diameter, 0.7 cm of thickness), were heat-treated in a furnace in order to burn out the PE powders and sinter the inorganic phase. The thermal treatment was set at a final temperature of 750 °C for 3 h. The heating rate was 5 °C/min. 

### 2.3. HA/BG_Ca Composites Characterization

Fine BG_Ca powders were first employed for the Differential Thermal Analysis (DTA). DTA (Netzsch DSC 404 differential thermal analyzer) was performed by using 30 mg of powders heated from room temperature to 1300 °C at 10 °C/min, in order to obtain the critical temperatures of the glass, such as the glass transition, crystallization and melting temperatures. 

The *in vitro* bioactivity of the HA/BG_Ca samples was investigated by soaking them in a simulated body fluid (SBF) solution, according to the standard protocol developed by Kokubo *et al*. [36]. SBF has inorganic ion concentrations similar to those of the human extracellular fluid. The samples were immersed in polyethylene flasks containing 20 mL of SBF and maintained at 37 °C. The SBF was refreshed every two days. After given times of 1, 3, 7 and 14 days the samples were extracted from the solution, rinsed with deionized water and then left to dry at room temperature. 

The sample microstructure before and after soaking in SBF was investigated by means of a scanning electron microscope, ESEM (ESEM Quanta 2000, FEI Co., Eindhoven, Netherlands). Moreover, a local chemical analysis was performed by X-ray Energy Dispersion Spectroscopy, EDS (Inca, Oxford Instruments, U.K.). The SEM was operated in low-vacuum mode with a pressure of 0.5 Torr.

The samples were also studied by means of X-ray diffraction (XRD). The samples were analyzed with a PANalytical X’pert PRO diffractometer employing a Cu kα radiation. Data were collected in the angular range 10–70° 2θ with steps of 0.017° and a scanning rate of 0.02° s^−1^.

### 2.4. Scaffolds Characterization

The scaffolds microstructure was investigated by means of a scanning electron microscope. Particular attention was devoted to the morphology, distribution and interconnection of pores. 

The total porosity (vol. %) of the scaffolds was evaluated by the following calculation

(1) $$P_{\%}=\left(1-\frac{W_{f}}{W_{0}}\right)\times 100$$

where *P_%_* is the total porosity (vol. %), *W_f_* is the measured weight of the scaffold and *W*_0_ is the theoretical one, calculated from the product of the HA/BG_Ca density by the scaffold volume. The density ρ = 2.99 g/cm^3^ of the composite was measured by means of a pycnometer test (Micromeritics AccuPyc 1330, Georgia, U.S.). Optical microscope images were also considered and analyzed with the aim to validate the porosity values. In particular, for each kind of scaffold, at least five images were acquired with an optical microscope equipped with a 10× objective. The area occupied by the pores was quantified by means of image analysis (UTHSCSA Image Tool).

Qualitative capillarity tests were performed on the scaffolds to assess their permeability, *i.e*., the presence of a highly interconnected pores network. To this aim, a solution with a viscosity (density ~1.05 g/cm^3^ [39], viscosity ~3.5 mPa·s [40]) similar to that of the human blood was used. Some drops of blue ink were dispersed into the solution to better observe the fluid infiltration throughout the scaffold. 

The mechanical resistance of the scaffolds was measured by means of compression tests (Zwick Roell Z600, equipped with an electronic estensimeter). The samples were indicatively 40 mm large and 7 mm thick disks. The load was applied along the thickness direction. The cross-head speed was set at 0.01 mm/min and the stress-deformation curve was recorded up to the maximum stress point. In particular, the failure stress *σ_f_* was defined as

(2) $$\sigma_{f}=\frac{L}{A}$$

where *L* (N) was the maximum applied load and *A* (mm^2^) was the nominal area of the cross section perpendicular to the load axis. For each type of material, five samples were considered to have statistical data. 

## 3. Results and Discussion

### 3.1. HA/BG_Ca Composites Characterization

**Figure 1:**
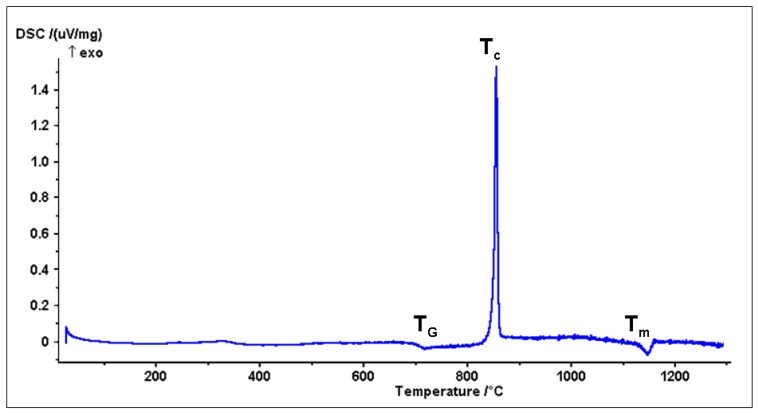
Differential thermal analysis of the BG_Ca glass (heating rate 10 °C/min).

Differential thermal analyses (Figure 1) indicated that the BG_Ca glass had a T_g_ at around 710 °C, an exothermal peak T_c_ at around 850 °C, and an endothermic peak T_m_ at around 1140 °C. T_c_ corresponds to the glass crystallization, while T_m_ may be attributed to the melting of the crystalline phase.

**Figure 2:**
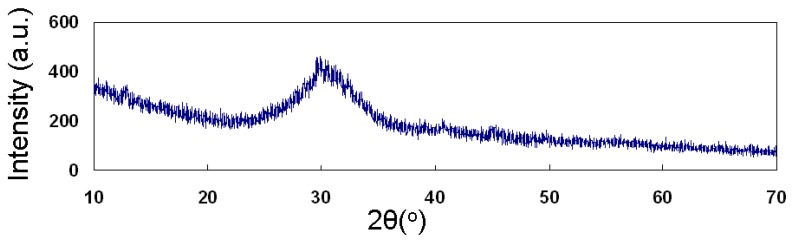
XRD spectra of the BG_Ca sintered sample treated at 750 °C for 3 hours.

Analogous studies regarding the widely used 45S5 Bioglass^®^ [41] report a lower crystallization onset temperature, at around 600 °C. The novel composition’s reduced tendency to crystallize compared to the 45S5 Bioglass^®^ can be attributed to its low sodium content. Crystallization, in fact, is regulated by two interactive factors: the viscosity of the glassy matrix and the diffusion coefficients of the ions which form the crystalline texture. Crystal nucleation and viscosity are both diffusion-related processes. Sodium is a glass-network modifier, capable of lowering the viscosity of silicate glasses by increasing the number of non-bridging oxygens [42,43]. This behavior can also favor crystallization. From this point of view, a decrease of all transformation temperatures—including the onset of melting—with an increase of the sodium content is reported in literature for different silicate-glass compositions [44]. Since the crystallization can slow down and hinder the kinetics of the sintering process, the BG_Ca powders are expected to consolidate at relatively low temperatures, which would be inadequate to confer a comparable compactness to a 45S5 Bioglass^®^ sample. Preliminary tests carried out on BG_Ca powders confirmed this property. The glass ability to maintain its amorphous nature up to very high temperatures is confirmed by the XRD analysis (Figure 2) performed on the BG_Ca sintered sample, treated at 750 °C for 3 hours. The pattern is characterized by a broad halo, indicating that the glass was completely amorphous.

**Figure 3:**
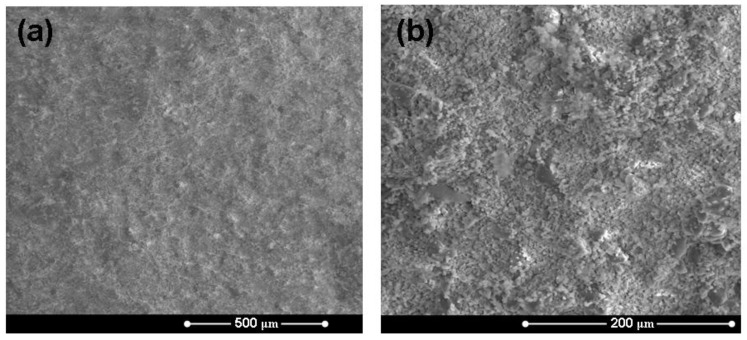
Micrographs of the surface of a HA/BG_Ca sample (different magnification degrees) treated at 750 °C for 3 hours.

**Figure 4:**
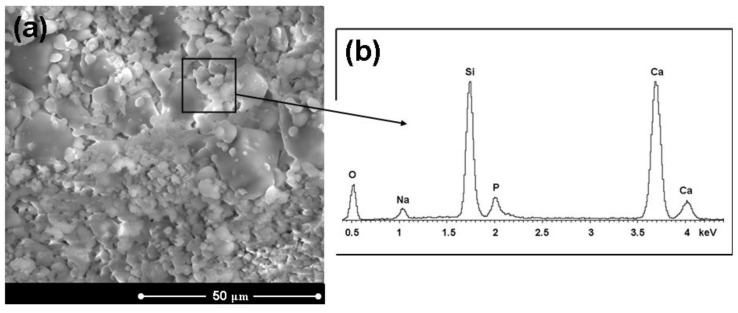
**(a)** Micrograph of a HA/BG_Ca specimen treated at 750 °C for 3 hours and **(b)** EDS results of the analysis carried out on the whole area reported.

Figure 3 reports some representative micrographs of the surface of a HA/BG_Ca specimen (sintered at 750 °C) at different magnification degrees. In general, the constituent phases are quite homogeneously distributed and the composite is well consolidated. Figure 4 shows a micrograph of a HA/BG_Ca specimen with the corresponding EDS spectra. The high Ca/Na ratio should be noted. 

**Figure 5:**
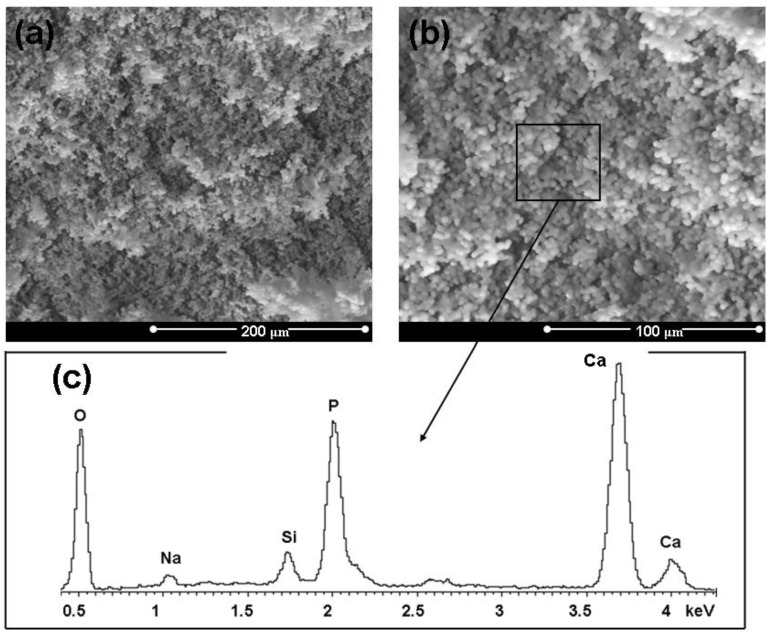
**(a, b)** Micrographs of a HA/BG_Ca sample (treated at 750 °C for 3 hours ) after 7 days in SBF and **(c)** EDS results of the analysis carried out on the whole area reported in **(b)**.

**Figure 6:**
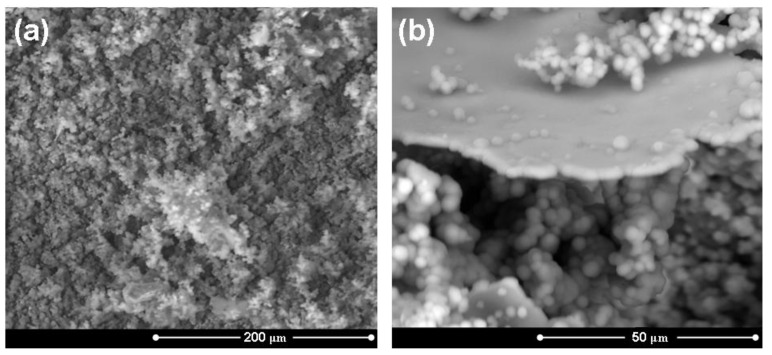
**(a)** Micrograph of the surface of a HA/BG_Ca sample (treated at 750 °C for 3 hours) after 14 days in SBF; **(b)** detail of the precipitated hydroxyapatite on the sample surface.

Great attention was paid to the *in vitro* behavior of the composites. In fact, since the formation *in vivo* of a HA layer at the interface between bone and implanted device is necessary for prostheses integration, the *in vitro* development of such a layer is usually considered as a fundamental preliminary requirement. Once immersed in a simulated body fluid solution, the composite starts its dissolution process. After soaking in SBF for 3 days, the composite is covered by a silica gel layer and it is possible to observe globular precipitates with the typical HA morphology, which are progressively growing and diffusing on the entire surface. After 7 days in SBF (Figure 5) the samples are entirely covered by HA. This fact is also confirmed by the EDS results reported in Figure 5, which show the presence of Ca and P in proportions similar to that of stoichiometric HA, since the Ca/P ratio is about 1.7 while in stoichiometric HA it is 1.67 [45]. Although Si is still present, as a result of the silica gel underneath the precipitates, its content appears lower because of the thicker HA layer above it. The HA precipitation has reached a much more advanced stage after 14 days in SBF (Figure 6). In fact, the original surface of the composite material is no longer recognizable. 

**Figure 7:**
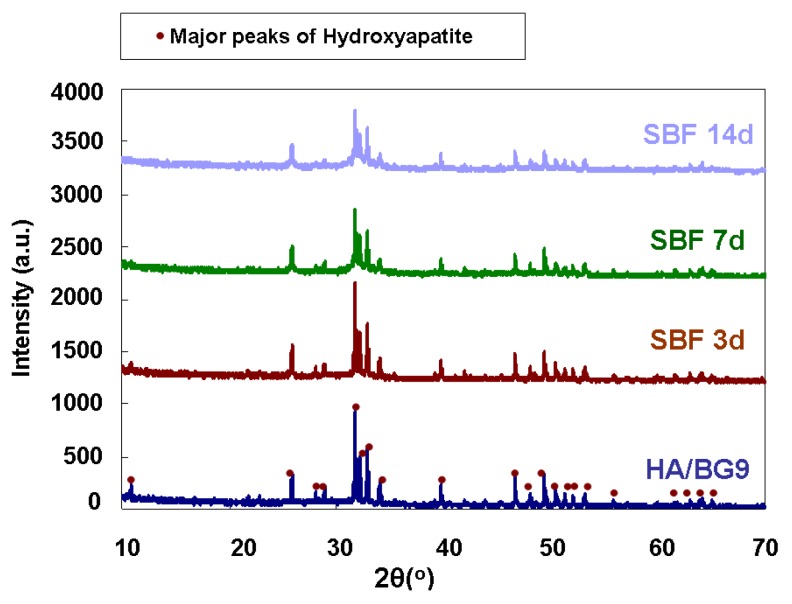
XRD spectra of the HA/BG_Ca composite treated at 750 °C for 3 hours before and after soaking in SBF.

The XRD analysis of the HA/BG_Ca composite before and after soaking in SBF is presented in Figure 7. The spectrum of the as-produced sample before and after immersion in SBF reveals only HA peaks; therefore the low temperature treatment minimizes the reaction between glass and HA. On the other hand, as reported in the introduction, literature regarding HA/bioactive glass composites often reports a complete reaction between glass and HA, resulting in an exhaustion, or at least a reduction, of the HA amount [27,30,46]. A slight broadening of the HA peaks with increasing soaking time is due to the micro-crystalline nature of the new HA precipitates [47]. On the basis of these interesting results, *in vivo* bioactivity of the HA/BG_Ca composite can also be expected.

### 3.2. Scaffolds Characterization

**Figure 8:**
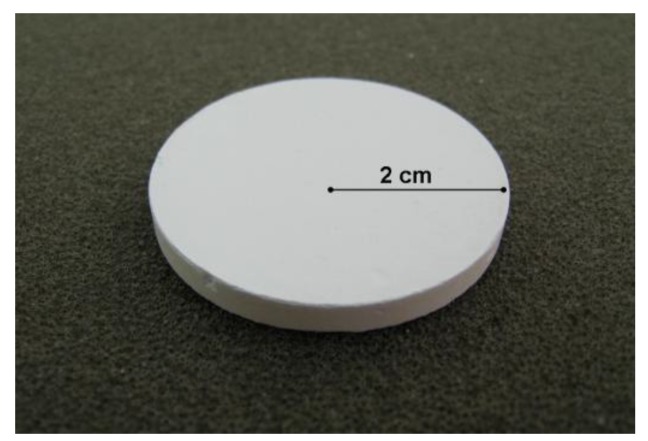
Digital image of a HA/BG_Ca scaffold treated at 750 °C for 3 hours.

Preliminary studies were focused on the feasibility of fabricating porous scaffolds with various PE contents. Several PE-to-composite HA/BG_Ca powders ratios were considered and it was found that after adding PE contents higher than 70 vol. % it was not possible to produce samples strong enough to be handled without damage. On the other hand, PE contents lower than 30 vol. % were insufficient to induce an adequate porosity [48]. In this study the microstructure of scaffolds realized using 50% vol. of HA/BG_Ca powders and 50% vol. of PE powders is offered as an example. The obtained samples are reported in Figure 8.

**Figure 9:**
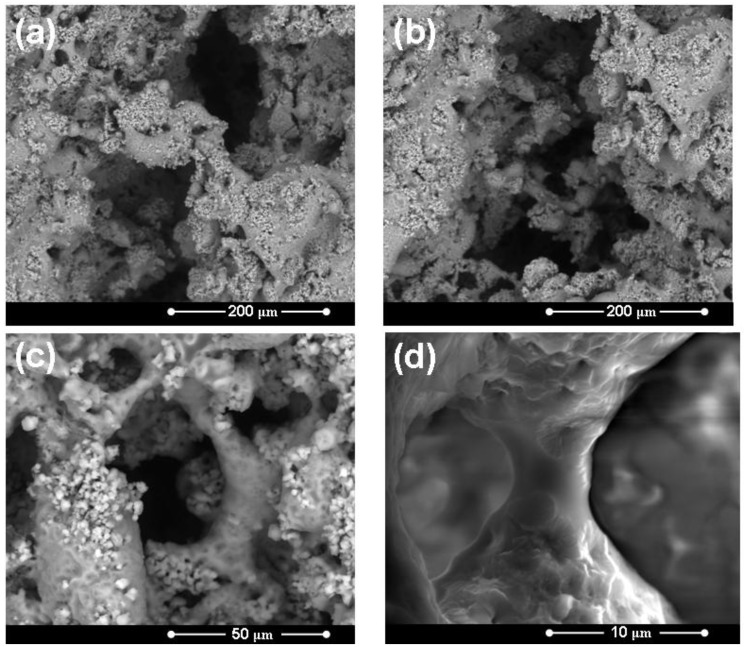
Micrographs of the HA/BG_Ca scaffolds microstructure: detail of the internal structure at different magnification degrees. The sample was treated at 750 °C for 3 hours.

Figure 9 shows the scaffold internal microstructure, revealing a distribution of large pores, as well as a high degree of interconnection between the pores. These features are crucial to foster an adequate vascularization and cell penetration *in vivo* [7]. It should be noted that the scaffolds were produced with mixtures of large (300–500 μm) and small (90–150 μm) PE particles. This is the reason why they exhibit not only interconnected macro-pores, but also a widespread network of micro-pores. The porosity is further augmented by the gas phases developed during the thermal decomposition of the PE spheres. The average pore size is greater than 100 μm, which is considered to be the minimum value required to allow bone cell infiltration and tissue in-growth [7]. In particular, in Figure 9(d) it is possible to observe that a good sintering level has been achieved and the struts between pores are well densified.

The average total porosity, calculated using Equations (1) on three samples is about 60 vol. %. This value was also confirmed by image analysis. However this technique, based on 2D images, is rather approximate, since the real porosity is intrinsically 3-dimensional. Preliminary samples obtained adding PE contents higher than 50 vol. % showed a higher porosity, reaching 70 vol. % (60 vol. % PE content), which is rather satisfactory for a burning out approach, especially in comparison to the results commonly reported in the literature, where typical values of 50 vol. %, or even less, are often reported [48,49].

**Figure 10:**
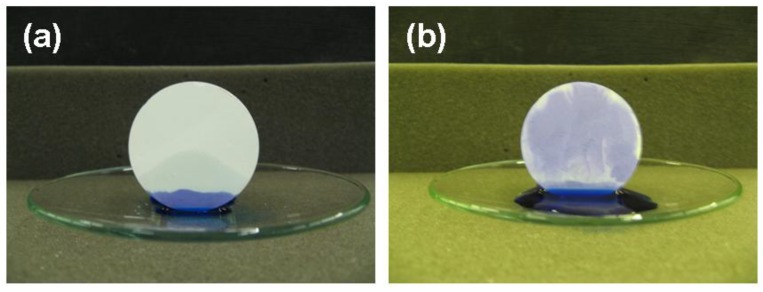
Images of the scaffold during the capillarity test. The sample was treated at 750 °C for 3 hours.

Figure 10 reports the outcomes of the capillarity tests performed on the produced samples in order to qualitatively investigate their permeability. A face of the scaffold was put into contact with the solution, previously colored by adding some drops of blue ink. The fluid infiltration within the scaffold, driven by capillarity forces, was immediately observed. 

**Figure 11:**
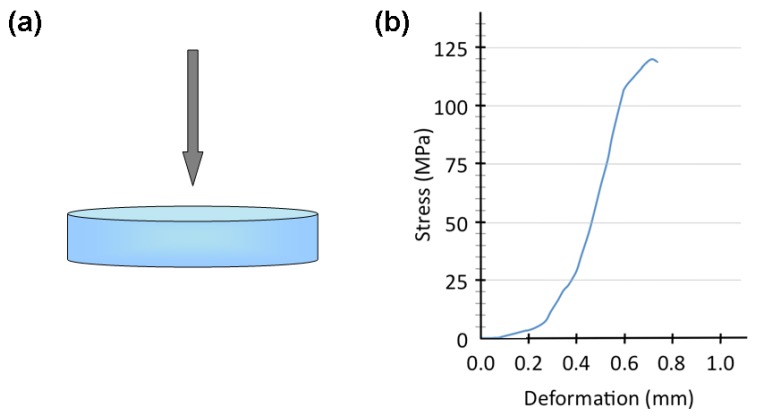
**(a)** Schematic representation of the compression tests layout. **(b)** Stress-deformation curve for a scaffold treated at 750 °C for 3 hours.

A typical stress-deformation curve for the obtained scaffolds is reported in Figure 11 together with a schematic representation of the compression tests layout. It is possible to observe a positive slope up to a peak point that identifies the failure stress *σ_f_* (see Equation 2). After that, an apparent stress drop occurs due to the collapse of the first struts. However it should be noted that the sample is still able to withstand higher loads, since the cracking phenomena at *σ_f_* only involves the weakest trabeculae among pores. From this point of view, this definition of the failure stress *σ_f_* is conservative, since it represents the limit of the Hookean-like region and not the complete breakdown of the scaffolds. The failure stress is 120.7 ± 2.1 MPa; this value is very high with respect to those usually reported in the literature [50]. However a similar behavior was reported by Baino and coworkers for high‑resistance scaffolds obtained via powder pressing and burning out [51]. In particular, they observed an anisotropic mechanical response of the samples, *i.e*., the scaffold resistance was superior if the load was applied along the compaction direction (as is the case in the present contribution) or along a transversal direction. This fact is probably due to the elongated shape of the pores, which resulted from the deformation of the PE particles caused by the pressure applied to the powders. Analogously, the high resistance observed during the mechanical tests performed in the present work could be attributed to a similar preferential orientation of pores. 

The porosity achieved in this feasibility study, the preserved amorphous nature of the glassy phase and the lack of HA thermal decomposition (and reaction) make the new HA/BG_Ca composite an intriguing system for the production of high-quality scaffolds. In particular, the possibility to employ this mixture to realize scaffolds by means of the widely used replication technique [4], which involves the production of ceramic foams by coating a polymeric sponge, is under investigation. 

## 4. Conclusions and Perspectives 

In this work, for the first time, glass-reinforced HA composites were produced employing BG_Ca, a glass composition belonging to the Na_2_O-CaO-P_2_O_5_-SiO_2_ system. This glass offers two basic advantages: it can be sintered at a relatively low temperature (750 °C) and it can preserve its amorphous nature up to 800 °C. In this way, the production of HA/BG_Ca composites is easier than standard HA/Bioglass ones. In fact, the HA/BG_Ca composites (in the present research: 50 wt % BG_Ca-50 wt % HA) can be sintered at 750 °C, while the standard HA/45S5Bioglass^®^ systems are usually treated at higher temperatures to achieve an adequate consolidation, typically in the 1150‑1300 °C range. Such drastic thermal treatments are likely to cause the HA to decompose and to cause the bioactive glass to crystallize into a glass ceramic. This fact, in particular, can potentially delay the bioactivity of the resulting samples. From this point of view the novel composites, which can be sintered without modifying the amorphous nature of the glass and the original composition of the HA, present a surprisingly high *in vitro* bioactivity. 

As a first application, the obtained composites were used to realize highly porous scaffolds by means of the polymer burning out method, which combines simplicity, versatility and low cost. The obtained samples are characterized by an open and interconnected pore network, with an average pore size higher than 100 μm. All the pores showed dense sintered struts. Such a microstructure provides adequate properties in term of permeability to fluids, verified by a capillarity test, together with an appreciable mechanical behavior according to compression tests. 

Further studies on HA/BG_Ca composites are under investigation, mainly regarding their *in vitro* degradation. In particular, it would be interesting to perform special immersion tests in SBF in order to evaluate the concentration of the elements released by the composites by means of inductively coupled plasma analysis. Moreover, in order to evaluate the effect of the immersion in SBF on the mechanical reliability of the composites, their microhardness will be measured before and after treatment; in the same way, compression tests will be performed on HA/BG_Ca-based scaffolds after different soaking intervals in SBF. Finally, the obtained results will be compared with the degradation behavior of BG_Ca-based scaffolds, with the aim to evaluate the effect of HA in the composite. 

The application of HA/BG_Ca composites to produce scaffolds by means of the replication technique is also in progress. Such a method, which reproduces the desired pore network directly in a ceramic matrix, overcomes the limits of the polymer burning out technique. 

Moreover, the new composite opens interesting scenarios for further applications in the development of prosthesis; in this sense, for example, it would be interesting to investigate the possibility to coat metallic substrates with HA/BG_Ca in order to make them bioactive. These results will be the subject of future studies. 
